# Electrospun Pt-TiO_2_ nanofibers Doped with HPA for Catalytic Hydrodeoxygenation

**DOI:** 10.1038/s41598-024-77103-4

**Published:** 2024-10-21

**Authors:** Amos Taiswa, Randy L. Maglinao, Jessica M. Andriolo, Sandeep Kumar, Jack L. Skinner

**Affiliations:** 1 Montana Tech Nanotechnology Laboratory, Montana Technological University, Butte, MT 59701 USA; 2 Advanced Fuels Center, Montana State University Northern, Havre, MT 59501 USA; 3 Department of Mechanical Engineering, Montana Technological University, Butte, MT 59701 USA; 4 Department of Civil & Environmental Engineering, Old Dominion University, Norfolk, VA 23529 USA

**Keywords:** Electrospinning, Nanofibers, Hydrodeoxygenation, Heteropoly acid catalyst, Bio-oil, Nanotechnology, Environmental sciences, Chemistry, Energy science and technology, Engineering, Materials science

## Abstract

Electrospinning is utilized to fabricate catalytic nanofiber scaffold for biocrude upgrading in hydrodeoxygenation (HDO) following computational studies suggesting the need for nano-catalysts for efficient HDO conversion and selectivity. Here, Pt-TiO_2_ nanofibers are fabricated through electrospinning, followed by wet impregnation with a heteropoly acid (HPA), tungstosilicic acid. Intensive heat treatments were incorporated during and after processes to obtain a HPA doped Pt-TiO_2_ nano-catalyst. Catalytic HDO was performed in a batch reactor with phenol as the raw biocrude dissolved in hexadecane. The HPA doped Pt-TiO_2_ catalyst demonstrated promising HDO performance of 37.2% conversion and a 78.9% selectivity to oxygen free benzene and the remainder 21.1% as diphenyl ester as a result of esterification by acidic components of the catalyst. Additionally, BET surface area characterization show a low surface area 16.9 m^2^ g^−1^ significantly lower than existing commercial catalysts and a mesoporous nature suitable for selectivity. The presence of HPA on the anatase nanofiber compensated for low platinum nanoparticles crystallinity on the nanofibers. This work might create needed alternatives for preparing HDO catalysts for efficient aromatics production.

**Supplementary Information:**

The online version contains supplementary material available at 10.1038/s41598-024-77103-4.

## Introduction

Production and consumption of sustainable fuels and chemical additives from biomass have continually increased due to increased climate change awareness and greater need for energy security.^1^ While biomass stands out as a promising carbon neutral energy source, it only accounts for 13 of global energy consumption.^2^ Biomass is the only renewable energy source that is rich with aromatics and fixed carbon that can serve as a raw material^3^ or provide a critical energy source for manufacturing practices. Each year, just 2% of the 70 million tons of aromatic rich lignocellulosic biomass produced from pulp and paper industries is converted to useful products worldwide.^4^

Deployment of clean energy technologies including biomass-based technologies could provide a substantial reduction in greenhouse gases (GHG) emissions.^5^ According to the Energy Information Administration (EIA), lignocellulose biomass contributes to over 50 of total renewable energy use^6^ The ease of access and abundance of biomass make biomass-based clean technologies an attractive path to lowering GHG emissions and achieving global carbon neutrality.^7^

Hydrothermal liquefication of biomass produces CH_4_ and CO_2_ gases, biochar, and bio-oil. These products are sources of alternative renewable, inexpensive, and readily available energy.^8^ Bio-oils produced from this process are abundant with oxygenated compounds such as ketones, oxyacid, furans, alcohols, and phenols.^9^ However, these products have undesirable properties, such as low heating values, high corrosivity, high viscosity and hydrocarbon fuel immiscibility.^10^

Catalytic hydrodeoxygenation (HDO) has been considered as a feasible approach to transform these oxygenated compounds into higher quality hydrocarbons.^11^ High-pressure HDO, which involves high hydrogen pressures and an active catalyst, is particularly effective in producing hydrocarbons from biooils.^12^ This process works by cleaving the carbon-oxygen (C-O) bond along with the saturation of the carbon-carbon double (C = C) bonds. While it successfully reduces the oxygen content in biooil, it also consumes a significant amount of hydrogen and destroys the aromatic structure due to hydrogen saturation. Aromatic hydrocarbons are essential in fuels for their lubricity, material compatibility, and role in creating sustainable fuel additives.^13,14^

The selective cleavage of C-O bonds to convert phenolic compounds found in biooils into aromatic hydrocarbons is still difficult. This step is crucial for transforming biooils into valuable chemical precursors for additives and lubricants. Despite extensive research, developing an efficient HDO catalyst remains a challenge due to the need of abundant hydrogen to penetrate active catalytic sites and lower the activation barrier energy of C-O bond cleavage.^15,16^

Recent experimental and computational studies using surface integrated molecular orbital/molecular mechanics (SIMOMM) theory have revealed a critical tendency: saturated hydrocarbons exhibited a propensity to move farther away from the catalytic metal sites.^17^ Therefore, a selective catalyst that can catalyze tautomerization and promote oxygen removal before molecules are saturated with hydrogen is of high importance for effective HDO. Nano-catalysts have shown promise in this essence with studies reported in Liu X et al. where Pt NPs were well dispersed in mesoporous silica for catalytic oxidation of CO by reducing 4-nitrophenol.^18^

Heterogeneous noble metal catalysts are commonly used for HDO, hydrogenation, and fuel cell catalysts.^19^ High cost and scarcity of precious metals has hindered their applicability at industrial scale.^20^ Metal catalysts often exhibit poor durability, low selectivity, and significant deactivation from coking and/or gas poisoning due to thermodynamic and diffusion limitations from biomass structures.^21,22^ Therefore, the need for inert ceramic catalytic materials that provide high surface area and porosity has grown over time.^23^ Electrospinning (ES) is an economically attractive fabrication technique for production of high surface area nanofibrous materials. Electrospun materials have provided good electron mobility and high photoelectric conversion efficiency due to high specific surface areas and high porosity, which creates efficient charge separation and transport.^24^ Fuel cells, nanogenerators, and enhanced photocatalytic hydrogen generators have been enabled through ES due to production of large surface area-to-volume ratio materials with improved crystallinity.^25^ In this work, ES was used to fabricate a high surface area catalytic scaffolding for HDO. The significance of this approach lies in the use of electrospun nanofibers, which provide multifunctional heterogeneous composites for processes that upgrade bio-oil, extending beyond just HDO. For instance, a ceramic membrane can be coated with crystalline catalytic heteropoly acids (HPAs) and/or noble metal nanoparticles (NPs) that can be reduced from respective salts^26,27^ Platinum compared to other common HDO metals such as palladium and ruthenium is less susceptible to catalyst poisoning by sulfur-containing compounds.^28^ HPAs have a high thermal stability and are suitable catalysts for harsh reactions.^29^

We report the fabrication and application of ES to produce scaffolds composed of TiO_2_ (anatase) and functionalized with Pt NPs for biofuel conversion. In post-processing methods, tungstosilicic acid crystals were incorporated into the scaffold structure via wet impregnation. Morphological properties and distribution of elements across the fibers were studied using field-emission scanning electron microscopy (FESEM) combined with energy-dispersive X-ray spectroscopy (EDS) and powder X-ray diffraction (XRD). Thermal stability and surface area measurements were performed to determine thermal and surface properties of the fibrous catalyst. Results presented here include testing of the functionalized biofuel-converting material in an HDO batch reactor.

## Results

### FESEM and EDS characterization of fibers

**Fig. 1:**
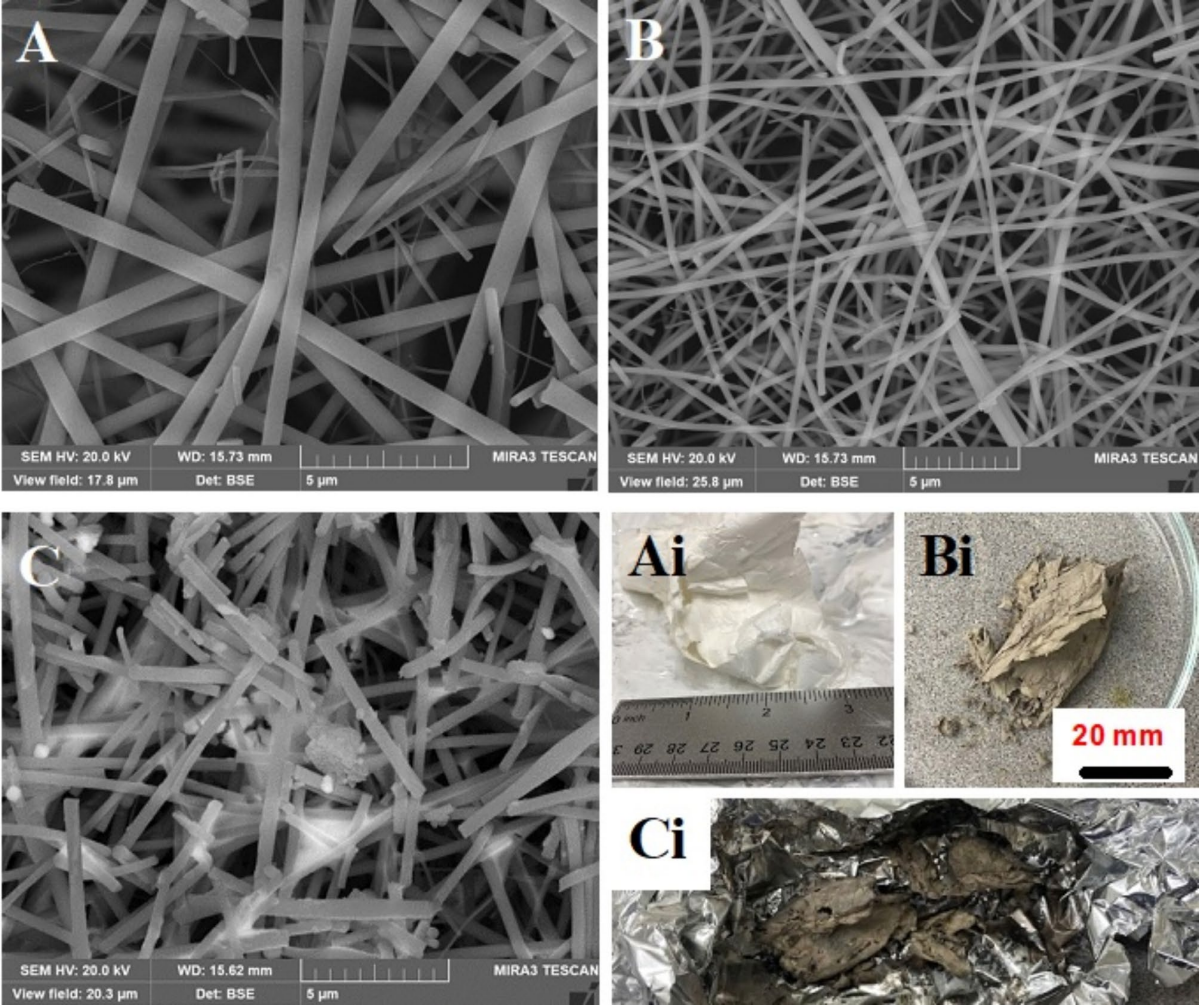
SEM micrographs and images of (**A**) electrospun PVP-Pt-TiO_2_ fibers with a picture insert (white color), (**B**) Pt-TiO_2_ fibers after calcining fibers in A at 550 ⁰C under nitrogen for 3 h with dark picture insert, (**C**) Pt-TiO_2_-HPA fibers calcined at 300 ⁰C, and (**D**) low magnification image of Pt-TiO_2_-HPA fibers with the EDS micrograph of the catalyst.

**Fig. 2:**
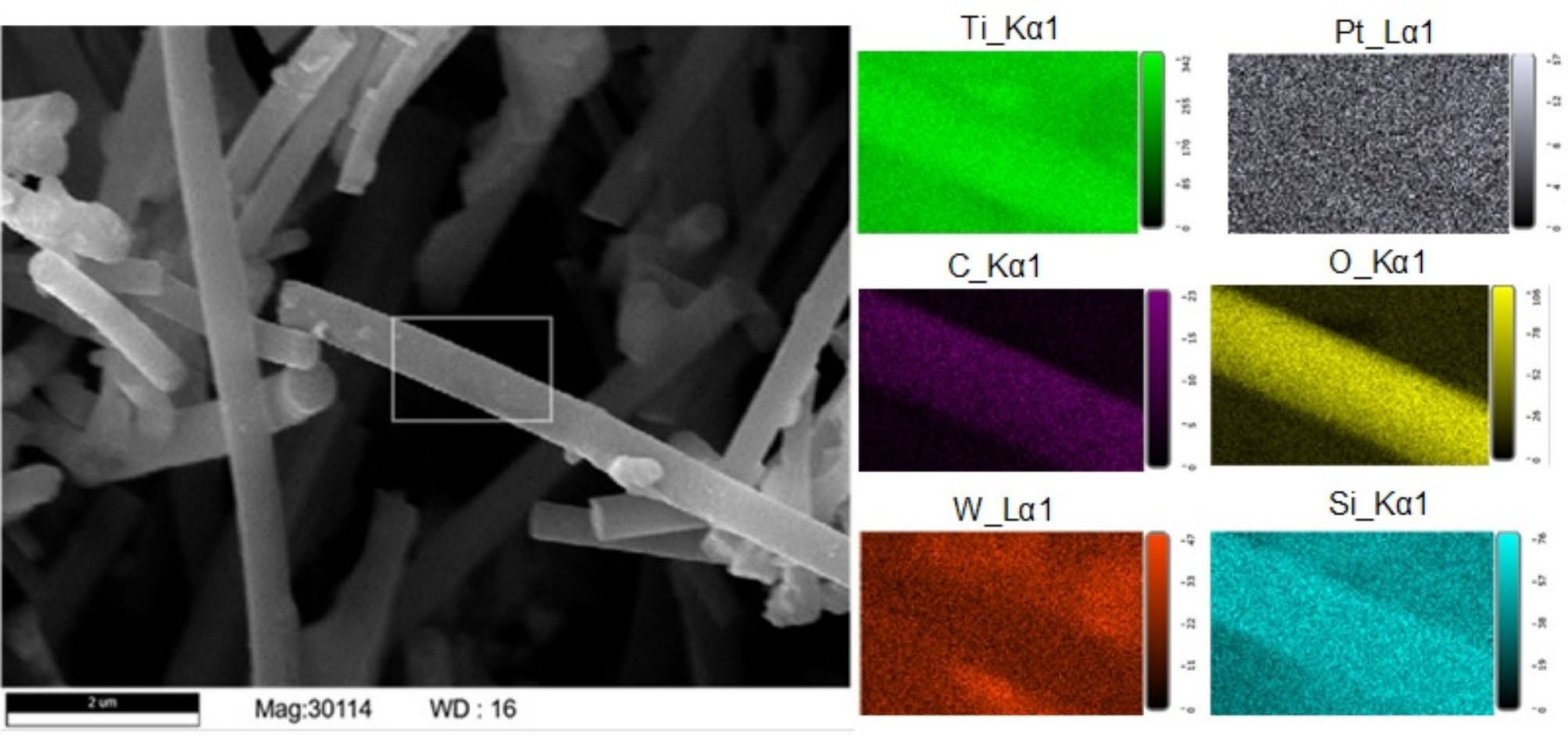
Distribution of Ti, Pt, C, O, W, and Si elements on a section of catalyst fiber.

The morphology and composition of PVP-Pt-TiO_2_, Pt-TiO_2_, and Pt-TiO_2_-HPA fibers were observed at different magnifications and are shown in Fig. 1A–D. Figure 1A, Ai shows an electrospun PVP-Pt-TiO_2_ fiber mat containing an average fiber diameter of 673 nm as determined with ImageJ image analysis. PVP has been reported to demonstrate desirable electrospinnability properties such as solubility in ethanol and acetic acid and excellent film forming ability.^30^ After calcining, the observed fibers were in crystalline Pt-anatase form that exhibited significant reduction in average fiber diameter and darkening of the fibers (391 nm, Fig. 1B, Bi). A large variety of fiber diameters observed in our samples was thought to be caused by varying environmental parameters, such as humidity and temperature. Such variation could be improved in future studies through control of these conditions. After calcination, Pt-TiO_2_ fibers exhibited traces of carbon that resulted from incomplete polymer and organic material combustion, commonly observed at moderate calcination temperatures. Figure 1C, Ci shows the fiber morphology after HPA deposition and calcination at 300 ⁰C. The post-calcination fiber mat had an average diameter of 337 nm with smaller fibers measuring 150 nm. The surface of the fibers became less smooth as the fabrication process continued as seen in Fig. 1A-C. The elemental composition of the electrospun catalyst showed the presence of key HPA elements (W, O, Si) as well as anatase and platinum in Fig. 2. As processing of the fibers in heat and solvents was performed, the fibers became more fragile (Fig. 1C).

Figure 2 shows the energy dispersive spectroscopy (EDS) of the electrospun Pt-TiO_2_-HPA fiber. The distribution of elements varied across the fiber depicted by the EDS map analysis. Vast segregation of Pt with concentrations spanning between 1.3 wt% and 3.3 wt% after calcination (pre calcination concentrations ranged 3.7 to 7.6 wt %) from EDS point, map, and line scan analysis (supplementary information **Fig. ****S1**** – S4**) suggests minimal grain growth from blending Pt salt in the polymer composite solution prior to ES (Fig. 2). Upon calcination, Ti content increased significantly from ~ 13 wt % to ~ 30 wt %, as TiO_2_ crystallinity increased significantly.

### X-ray diffraction (XRD)

Pt crystals were not identified in the XRD, possibly suggesting a low concentration on the Pt-TiO_2_ fiber and final Pt-TiO_2_-HPA catalyst. Pt-TiO_2_ fiber matched peaks for anatase as standardized by PDF 98-001-4287 library source shown in the supplementary information **Fig. S5**. HPA peaks matched those of H_4_W_12_SiO_40_⋅6H_2_O (PDF 00-001-0559). Overall peaks of Pt-TiO_2_-HPA were similar to that of the purchased HPA suggesting no breakdown of the HPA to tungsten and silicon oxide constituents or possible secondary phases from catalyst components reacting after 300 ⁰C sintering. The tendency of HPAs to disintegrate into respective constituents is discussed in detail by Zhang et al.^31^ Anatase fibers were able to maintain structure throughout the fabrication process as evidenced by the presence of its XRD peaks in the final catalyst. The final XRD diffractogram showed no Pt peaks and a pureTiO_2_-HPA composition.

### N2 physisorption method

Specific surface area of these fibers was investigated using nitrogen adsorption measurements (Table 1) and was found to be 16.9 m^2^ g^−1^. The N_2_ adsorption/desorption isotherms demonstrated a mesoporous characteristic (majority of the pore sizes range from 2 to 50 nm) which plays a significant role in selectivity by facilitating the diffusion of reactant molecules to active sites within the catalyst.^32^ The interconnected network of mesopores allows for efficient mass transport of reactants and products, reducing diffusion limitations that can hinder catalytic performance enhancing selectivity by ensuring that reactant molecules reach the active sites more effectively.^33^ Pt-TiO_2_-HPA fibers had specific surface areas similar to that observed PVDF (polyvinylidene fluoride) nanofiber membranes reported in an earlier study by Fenlin et al.^34^ The specific surface area of electrospun fibers was compared to those of commercially available palladium on activated carbon (Pd/C) and ruthenium on zinc oxide (Ru/Zn_2_O_3_) catalysts and was generally large in comparison to commercial Pd/C (1217.7 m^2^ g^−1^) and Ru/Zn_2_O_3_ (165.3 m^2^ g^−1^).

The key advantage of the final electrospun catalyst was that is required a low hydrogen operating pressure. Low hydrogen pressure will be required in a conventional hydrodeoxygenation process to reach the larger pores of Pt-TiO_2_-HPA fibers. Surface area of the fibers can be increased significantly by reducing the fiber diameters during ES.

**Table 1:** BET surface area comparison of the electrospun fibers to conventional commercial catalysts.

Pore size	ES Pt in TiO_2_-HPA Catalyst, m^2^ g^−1^	Pd on Activated C Catalyst, m^2^ g^−1^	Ru on Zn_2_O_3_ Catalysts, m^2^ g^−1^
S_BET_	16.9	1217.7	165.3
< 2 nm	0.3	905.3	0.0
Between 2–50 nm	8.6	311.9	165.1
Between 50–100 nm	8.0	0.5	0.2

### TGA-MS

**Fig. 3:**
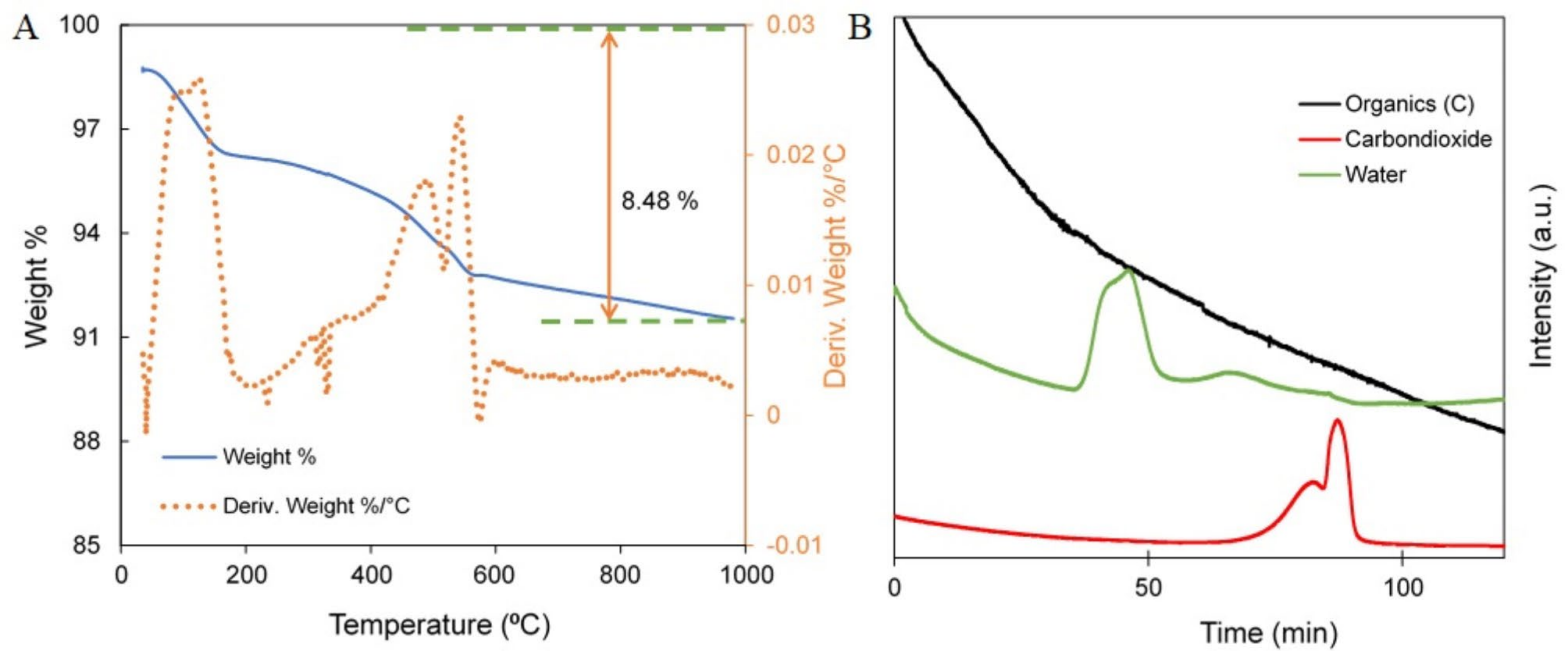
(**A**) TGA thermogram of the electrospun catalyst. Heating rate at 10 ⁰C/min up to 1000 ⁰C under Ar. (**B**) MS graph of effusing gases from the thermally combusting catalyst.

The thermal stability of the catalyst was examined by conventional weight loss upon linear increase of heat by the TGA. Pt-TiO_2_-HPA was maintained at room temperature for 10 min to normalize the ion filament and gasses followed by a steady ramp of 10 ⁰C until a final temperature of 1000 ⁰C was achieved. Figure 3A shows the temperature dependent weight loss of the catalyst. The TGA curve shows a steep weight loss between 35 ℃ and 180 ⁰C traced by the MS as a point of high moisture loss and carbon combusting off as CO_2_ and organics/carbon-14 (Fig. 3B). Derivative TGA show more thermal steady decomposition of the catalyst at 610 ⁰C. The MS integration shows organics, carbon dioxide, and moisture contributed to the observed 8.48% weight loss. The absence of key HPA and TiO_2_ elements in MS detection provided evidence of the thermal stability of the material.

### Catalysis performance

Following the catalytic HDO of phenol using the electrospun catalyst, GC-MS analysis confirmed the presence of both benzene and phenol in the final product. The catalyst exhibited a mere 37.2% conversion of phenol producing benzene and diphenyl ether. However, the successful addition of the HPA onto the TiO_2_ fibers played an important role, resulting in 78.9% selectivity toward benzene and 21.1% diphenyl ether. Interestingly, similar trends have been reported in other studies. Nelson et al.^35^ reported a conversion rate and selectivity toward benzene of 30%and 95% respectively, over Ru/TiO_2_. De Souza et al.^36^ observed 7.0% ad 66.8% cnversion and selectivity, respectively, over Pd/TiO_2_ catalyst. Both of these studies also reported the formation of other byproducts, such as cyclohexane and cyclohexanone, which were not observed in our study. Performances under different catalysts are summarized in Table 2 below.

**Table 2:** Resume of some catalytic HDO performances in literature using different catalysts with unique surface area. (where zero benzene selectivity was recorded, phenolic monomers were completely saturated to hydrocarbons like toluene and cyclohexane).

References	Catalyst	Area (m^2^.g^−1^)	Conversion %	Benzene Selectivity, %
Nelson et al.^35^	Ru/TiO_2_	33–55	30	95
De Souza et al.^36^	Pd/TiO_2_	54	7	67
Zhang et al.^37^	Ru/Nb_2_O_5_-MC	290.97	7	14
Boullosa-Eiras et al.^38^	Mo_2_C/TiO_2_	35–74	13–30	60–90
Resende et al.^39^	Pd.n./ZrO_2_	73– 79	7.7–13.5	7.5–41
Li et al.^40^	Pt/TiO_2_	291	33–100	0
Yu et al.^41^	Ni_2_P	3.9	80	0
**This Work**	**Pt/TiO**_**2**_ **-HPA**	**16**	**37**	**79**
Wu et al.^42^	Pt/TiO_2_	110	92	0

**Fig. 4:**
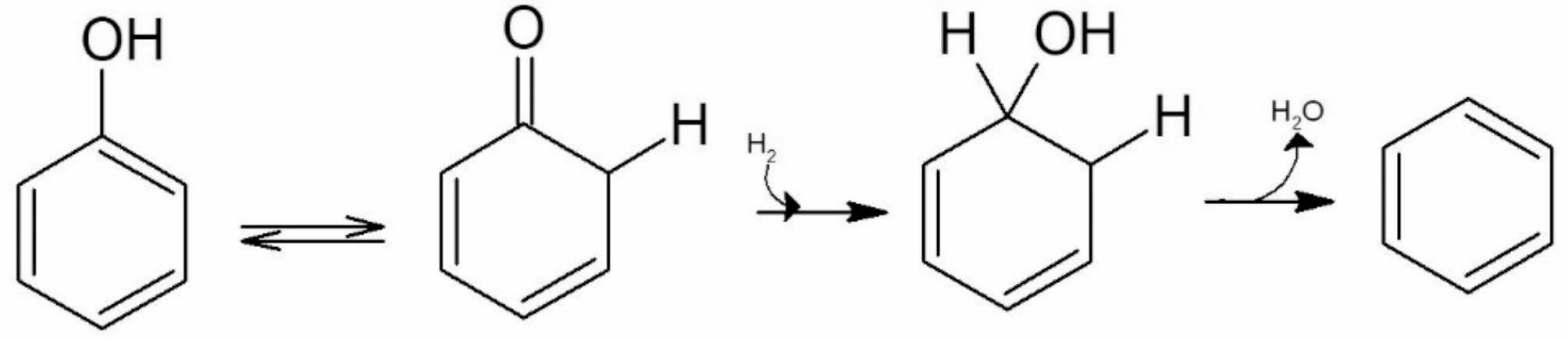
Keto-tautomer pathway of phenol to benzene over H_2_ at 4.14 MPa and 250 ℃. Phenol initially isomerizes to its keto tautomer before hydrogenation and subsequent dehydrations.

According to the hypothesized keto-tautomer mechanism proposed by de Souza et al.^36^ and referenced by Maglinao et al.,^14^ phenol HDO involves several sequential steps. Initially, phenol isomerizes to its keto tautomer, which can occur in solution or on the Pt surface. Subsequently, the carbonyl group undergoes hydrogenation on the Pt surface. The formation of benzene in this study suggests that phenol isomerized into its keto form under the catalyst (Fig. 4), with the –OH group being cleaved without saturating the aromatic ring.^43^ The authors also hypothesized that the limited amount of Pt on the catalyst hindered the initial hydrogenation step necessary for the HDO process. This aligns with the findings of Zanutti et al., who observed that higher Pt loading favors the hydrodeoxygenation of *m*-cresol to toluene, while lower Pt loading results in slower toluene formation.^44^ The observed low conversion rate is directly attributed to the scarcity of Pt on the catalyst.

**Fig. 5:**
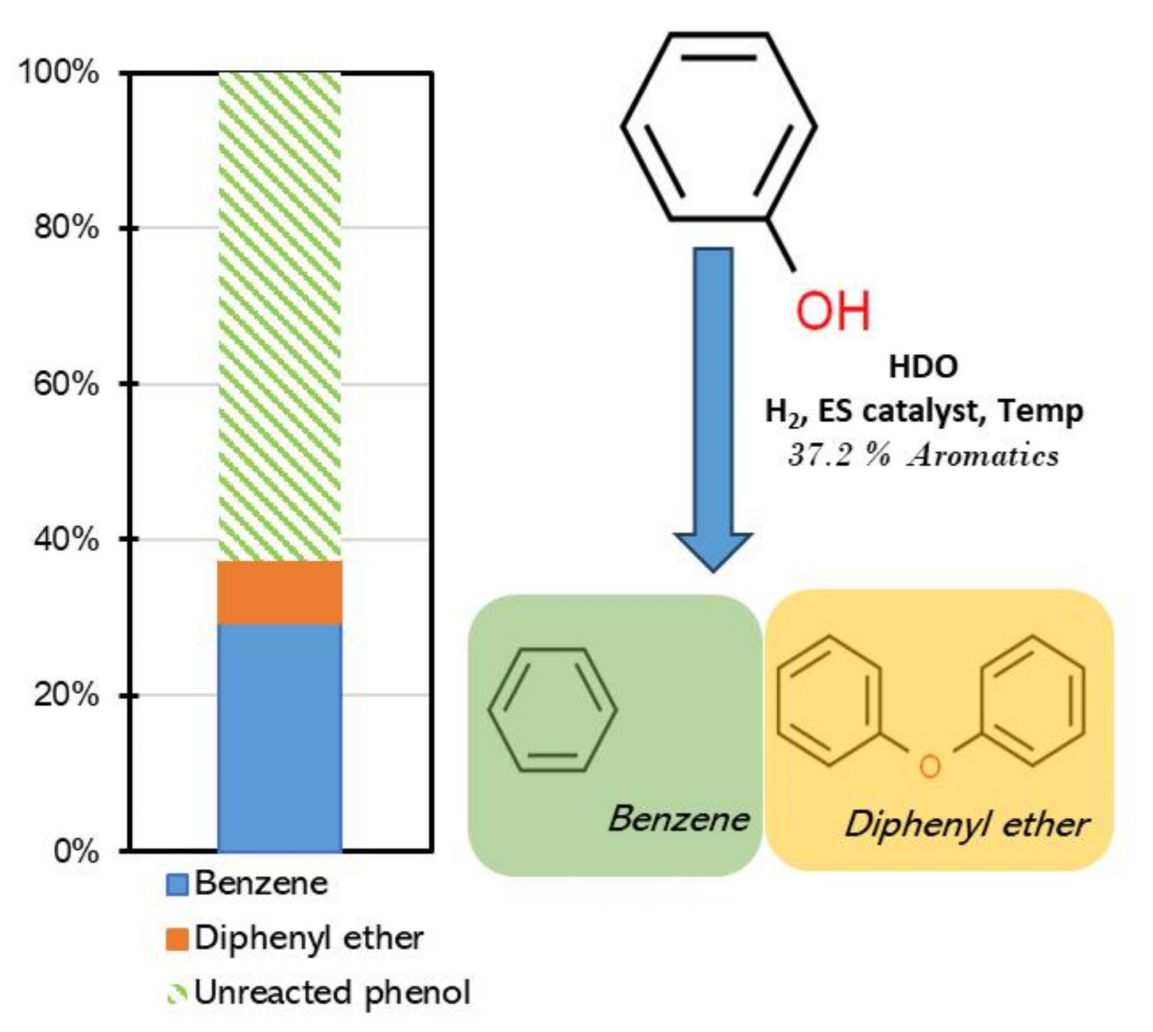
Conversion mechanism observed under the electrospun catalyst. Phenol was converted to benzene and some to diphenyl ether which resulted from esterification of phenol by acidic components of the catalyst.

The cyclohexadienol intermediate, formed during hydrogenation of the carbonyl group (Fig. 4), undergoes dehydration over the TiO_2_ support a known active site for alcohol dehydration^45,46^. In this study, the HPA introduced an additional site for dehydration, facilitating the immediate formation of benzene after the initial hydrogenation step. The absence of cyclohexanone in the product further supports this observation. With more sites available for dehydration than hydrogenation, the competing hydrogenation reaction leading to cyclohexanone is unlikely to occur. This is supported by studies indicating that HPA-modified supports contain additional acid sites that aide C-O bond cleavage during hydrodeoxygenation.^47,48^ Furthermore, employing density functional theory, theoretical calculations of the phenol HDO mechanism revealed that HPA effectively reduces the activation energy of deoxygenation during HDO reactions.^49^ Overall, the TiO_2_ support and addition of HPA enhanced the overall selectivity of the catalyst toward benzene formation (Fig. 5).

Choosing an alkane, such as hexadecane, as the solvent in the phenol HDO favors the initial hydrogenation of the carbonyl group over hydrogenation of carbon-carbon double bonds within the aromatic structure. This preference arises because nonpolar compounds are less likely to interact with the lone pairs in the carbonyl group.^14^ Although hexadecane contributed in the HDO of phenol, the limited Pt in the catalyst hindered the initial hydrogenation reaction step, which resulted in low phenol conversion.

**Fig. 6:**
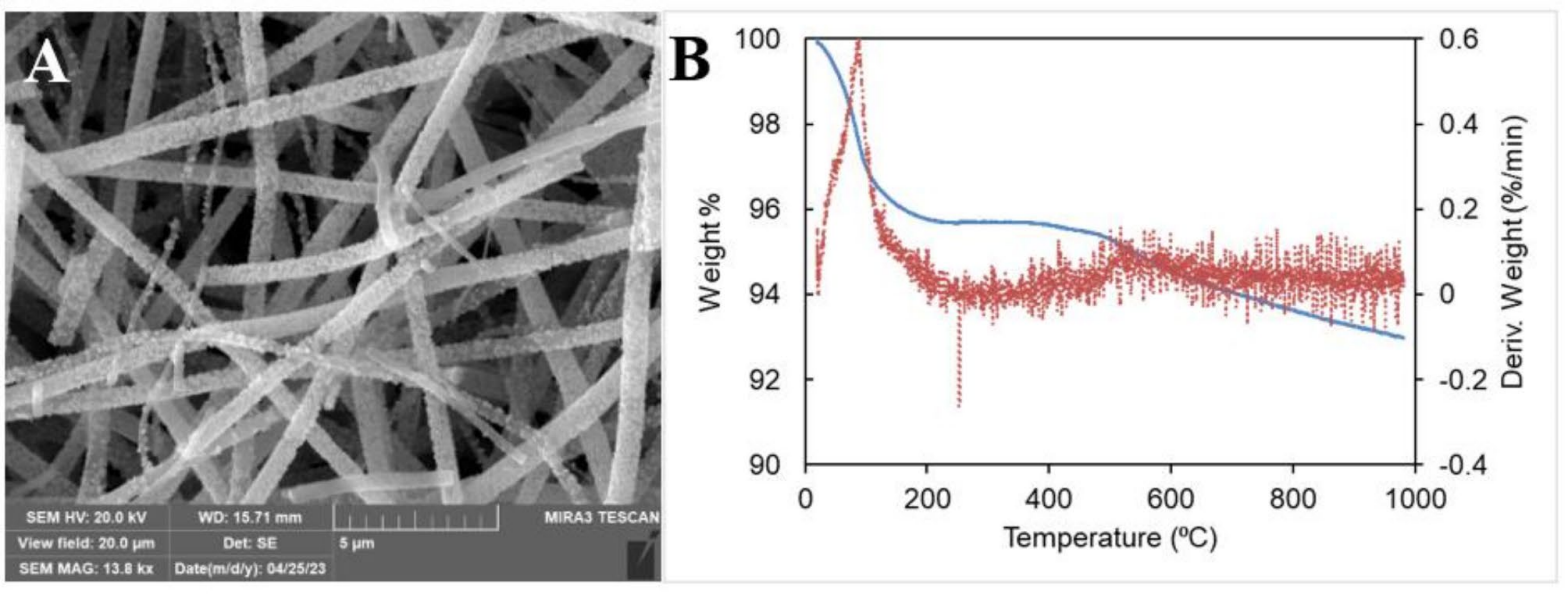
SEM of the Pt-TiO_2_-HPA catalyst after use with its representative EDS point analysis. (B) TGA microgram of the used catalyst after a 10 ⁰C ramp rate from room temperature to 1000 ⁰C.

The post HDO catalyst was engulfed with HDO reactants as shown in Fig. 6A. The TiO_2_ fiber exhibited thermal stability (Fig. 6B) and maintained physical structure during the reaction process as confirmed by the EDS point analysis data in supplementary **Fig. S10**. These two features of the electrospun catalysts make it a promising alternative with potential reusability. It can be noted that Pt crystals were not detected on the used fiber, hence, reusing the catalyst necessitates regeneration of Pt. After HDO, there is significant amount of organic debris on the catalyst due to this can be evidenced by high mass loss in thermographic analysis in Fig. 6B between room temperature and 200⁰C compared to a pre-HDO catalyst in Fig. 3A.

## Discussion

Although this work is performed at a reduced overall catalytic conversion rate due to the absence of high crystallinity in Pt NPs (Fig. 2), the electrospun nanofiber catalyst clearly showed potential as a catalyst. High scalability potential of ES and vast alternatives of growing crystalline NPs and nanowires on the fiber surface has previously been performed. The electrospun catalyst did not only successfully convert phenol to 78.9% oxygen free benzene and 21.1% diphenyl ether, but it also promoted the adhesion of HPA onto the fiber. Thermal and bonding stability of catalyst components on the fibers improves its recovery and reduces leaching of elements from the fiber. Improving metal concentration and homogeneity has been studied extensively for instance, Wang et al.^50^ and Newman et al.^51^ show that increasing the metal concentration, Ag/TiO_2_ and Ru/TiO_2_ respectively, results in the creation of oxygen vacancies (Ov). These Ov are deoxygenation sites and in both cases a; linear relationship between metal loading and conversion % was recorded.

**Fig. 7:**
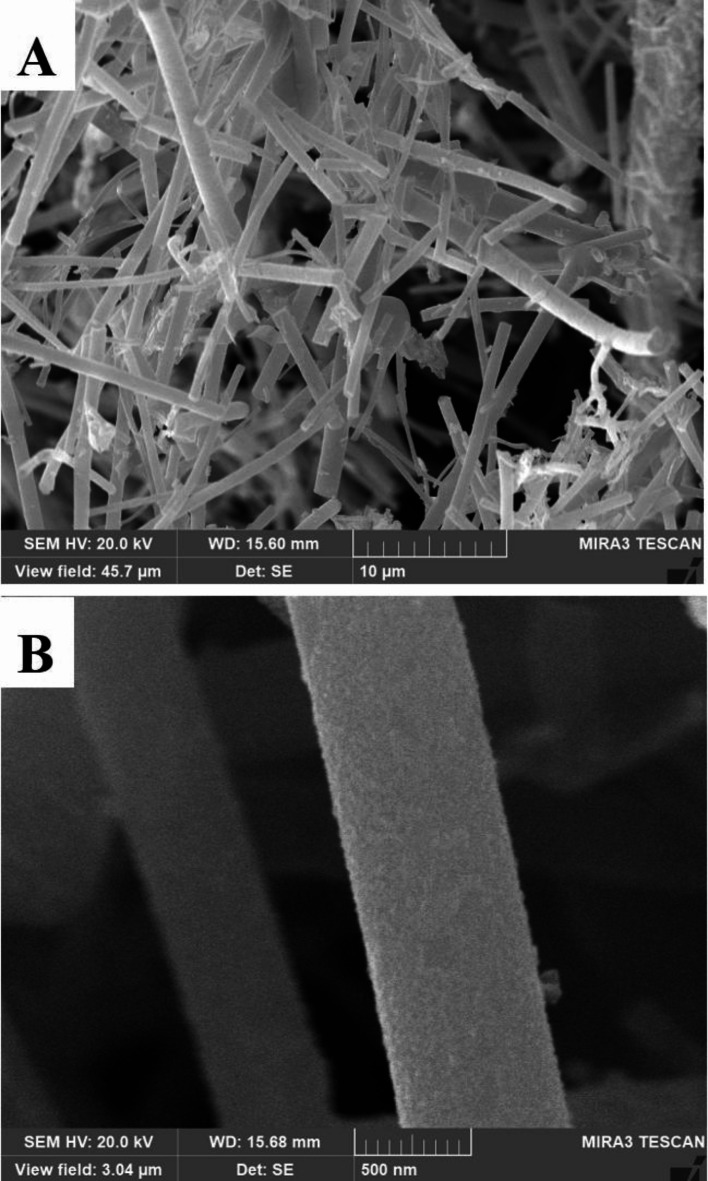
SEM of a TiO_2_-HPA fiber decorated with Pt NPs through a polyol process at (A) wide view and (B) detailed nanofiber decorated with NPs.

Independent of this study, the ES process was modified to investigate growth of Pt NPs on electrospun anatase fibers. To achieve these structures, the fibers were electrospun from a titanium precursor in a PVP-ethanol solution. The fibers were then calcined under nitrogen atmosphere conditions before impregnating them with HPA. Pt NPs were deposited on the final catalyst by a polyol reduction bath. The final catalyst was filtered out and rinsed with deionized water. Using this method, higher Pt concentrations were achieved (Fig. 7) than when embedding Pt in the anatase fibers (Figs. 1 and 2). Such a method will be employed in future studies to improve catalytic performance.

Fabrication of an HDO catalyst consisting of a TiO_2_ scaffold, Pt NP functionalization, and tungstosilicic acid crystals for biofuel conversion was accomplished. The fabrication methods used included ES and calcination steps that were simple and scalable. Characterization of the composite nanofiber catalyst revealed smooth fiber morphologies with an average fiber diameter of 337 nm. XRD and EDS measurements revealed the expected crystal structure and composition, and TGA-MS was used to verify a preferrable thermal stability of the material. The catalyst was reduced in H_2_, performance was tested on a batch reactor, and benzene selectively was measured at 78.9% without saturating the rings. Negligible presence of Pt on the catalyst resulted in a 37.2% conversion of phenol, whereas the successful addition of the HPA on the TiO_2_ fibers successfully played an important role in a 78.9% benzene selectivity. Such results demonstrate great promise that the ES and post-processing methods used may be repeated and improved to produce a high surface area, efficient, biofuel converting material.

In future work, we will aim to improve consistency of fiber morphology through environmental controls, evaluate the catalyst through HDO, scale up the fabrication process, and optimize metal concentrations to the catalyst scaffold to further enhance biofuel converting efficiencies of the catalyst.

## Materials and methods

### Synthesis of nanofibers

Platinum-titanium dioxide (Pt-TiO_2_) nanofibers were prepared from a solution containing 40 mg of chloroplatinic acid (H_2_PtCl_6_), 3 mL titanium tetraisopropoxide (C_12_H_28_O_4_Ti) dispersed in a 6 wt % poly(vinylpyrrolidone) (PVP, M_w_ ≈ 1.3 × 10^6^) in a 1:2 acetic acid to ethanol mixture. The solution was stirred overnight (~ 12 h) at room temperature until all the solids dissolved to form a clear golden gel solution. The solution was then loaded onto a vertical electrospinner equipped with a voltage source, collection plate, 20-gauge spinneret, and syringe pump. A high-power supply operating at 10.8 kV was connected to the spinneret and collector. The separation distance between the spinneret and collector was maintained at 6 cm, and the propulsion rate continuously held at a rate of 0.75 mL/h. The electrospun fibers were deposited onto an aluminum foil and were /-left overnight (~ 12 h) to fully hydrolyze in air. The composite fibers were then calcined in nitrogen at 550 ℃ for 3 h to remove the PVP.

### Tungstosilicic acid deposition on Pt-TiO_2_ nanofibers

A wet impregnation technique was used to coat the fibers with (H_4_W_12_SiO_40_·6H_2_O) HPA. In this method, calcined fibers were soaked in 20 wt % HPA in ethanol solution for 12 h. Ethanol was then drained, and the fibers dried for 2 h at 120 ℃ followed by a 3 h calcination in air at 300 ℃ to strengthen HPA onto the fibers without breaking structure as discussed by Zhang et al.^31^

### Catalytic testing

HDO reactions were conducted in 20 mL T316 stainless steel pressure reactors from Parr Instrument Company (Moline, IL). A Parr 4838 temperature controller was used to regulate and monitor reaction temperature. Phenol (≥ 99%, Acros), and *n*-hexadecane (99%, Acros) were used as received throughout the experiments. The catalyst was first reduced at 200 ⁰C for 2 h under pure hydrogen gas at 4.14 MPa, and then cooled down to room temperature overnight. Once cooled, excess hydrogen gas was released. A glass syringe was used to inject ca. 5.4 g of hexadecane into the closed reactor while the catalyst was still inside. Submerging the catalyst in hexadecane prevented the reduced catalyst from reacting with atmospheric oxygen. The reactor was dissembled, and 2.4 g of phenol was added to the mixture. Hydrogen gas was used to purge the reactor after reassembly. The mixture was reacted at 250 ⁰C for 6 h under hydrogen gas held at 4.14 MPa.

### Characterization

A Tescan Mira 3 FESEM connected to an EDS (Ametek) at an accelerating voltage of 25 kV was used to characterize the morphologies and elemental composition of hydrolyzed and calcined Pt-TiO_2_ fibers and Pt-TiO_2_-HPA before and after catalytic testing. The crystal phases of HPA as purchased, calcined Pt-TiO_2_, and Pt-TiO_2_-HPA powder and NFs were investigated with XRD (Rigaku IV) using cobalt radiation source at 40 kV and 40 mA with scans taken in Bragg-Brentano geometry between 10⁰ and 70⁰ 2θ, cobalt Kα radiation, with a wavelength of 1.7902 Å, is characterized by its longer wavelength in contrast to copper Kα radiation, which has a wavelength of 1.5406 Å, hence deeper sample penetration. Thermal stability and volatile composition of the final catalyst was analyzed using thermal gravimetry coupled with mass spectrometry (TGA-MS). For TGA-MS, samples were heated in ultra-pure argon in an evolved gas analysis (EGA) furnace on a thermal gravimetry analyzer (TGA; TA Instruments). Ion current for daltons (amu) of ionized decomposition species was recorded using a mass spectrometer (Thermostar GSD 350; Pfeiffer Vacuum) equipped with a quartz capillary gas inlet connected to the EGA furnace. Brunauer-Emmett-Teller (BET; Nova 800, Anton Paar) specific surface area and porosity analysis were performed using low temperature (77 K) nitrogen physisorption method over a wide range of relative pressures from 0.002 to 1. The samples collected after HDO were analyzed using a gas chromatography-mass spectroscopy (GC-MS) to detect the presence of oxygen free aromatics.^14^ The computational chemistry calculations were performed using an open-source quantum chemistry software General Atomic and Molecular Electronic Structure System (GAMESS) with SIMMOM approach used to investigated the HDO of phenol in the presence of catalytic constituents.^52,53^

## Electronic supplementary material

Below is the link to the electronic supplementary material.

Supplementary Material 1

## Data Availability

The datasets used and/or analyzed during the current study available from the corresponding author on reasonable request.
