# Supplementary material for: Electrospun Pt-TiO2 nanofibers Doped with HPA for Catalytic Hydrodeoxygenation
Source: Sci Rep. 2024 Oct 21;14:24706. doi: 10.1038/s41598-024-77103-4 (PMC11493970; doi:10.1038/s41598-024-77103-4)
Supplement: Supplementary file 1 — Supplementary Material 1 [file 41598_2024_77103_MOESM1_ESM.pdf]

Supplementary Information

Electrospun Pt-TiO_2 Nanofibers doped with HPA for Catalytic Hydrodeoxygenation

Amos Taiswa,* [1,3] Randy L. Maglinao,[2] Jessica M. Andriolo,[1,3] Sandeep Kumar,[4] and Jack L. Skinner[1,3]

[1] Montana Tech Nanotechnology Laboratory, Montana Technological University, Butte, MT 59701

[2] Advanced Fuels Center, Montana State University Northern, Havre, MT 59501

[3] Department of Mechanical Engineering, Montana Technological University, Butte, MT 59701

[4] Department of Civil & Environmental Engineering, Old Dominion University, Norfolk, VA 23529

*Corresponding author: ataiswa@mtech.edu

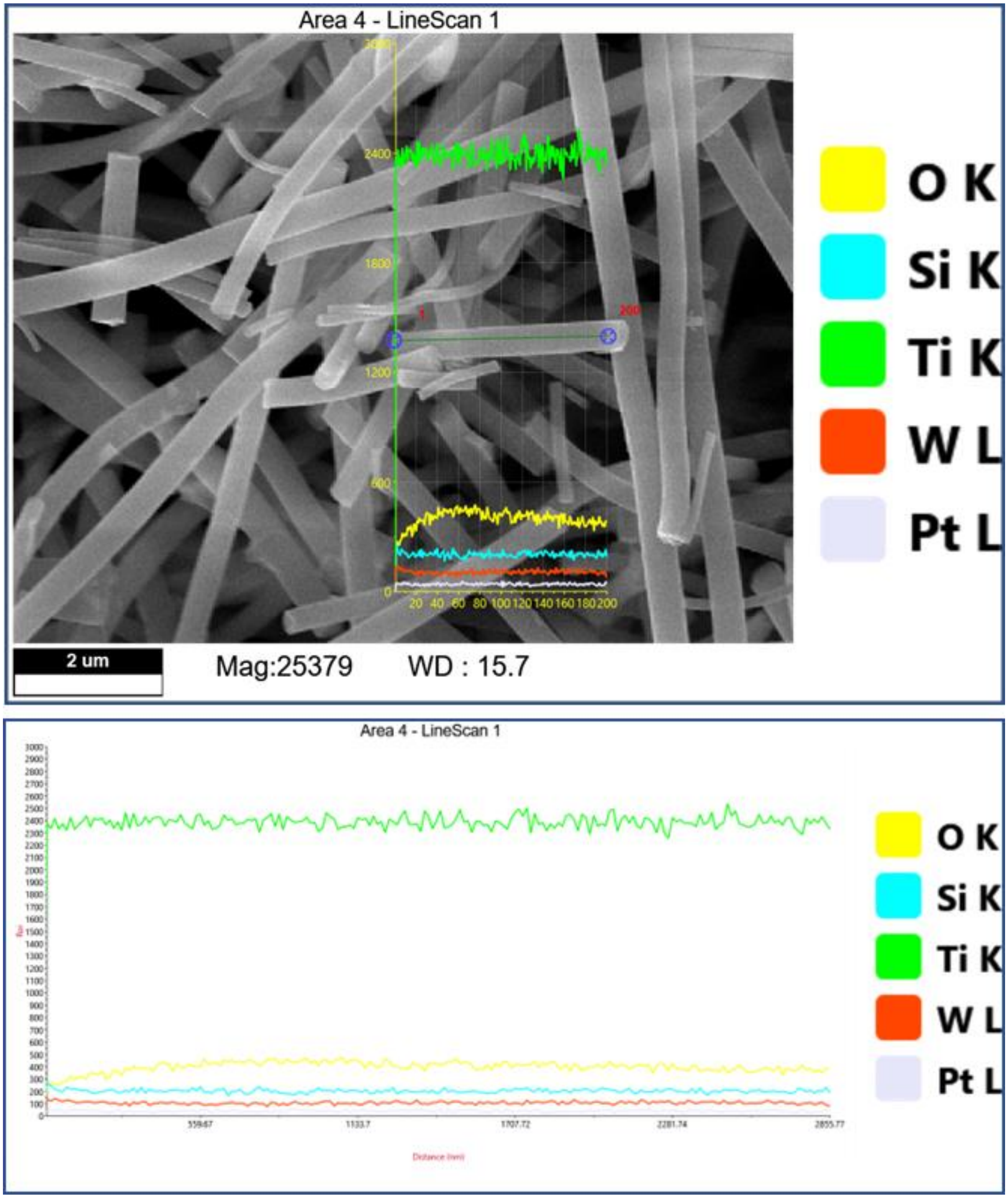

Fig. S1: EDS line scan of a random fiber in the Pt-TiO_2-HPA catalyst.

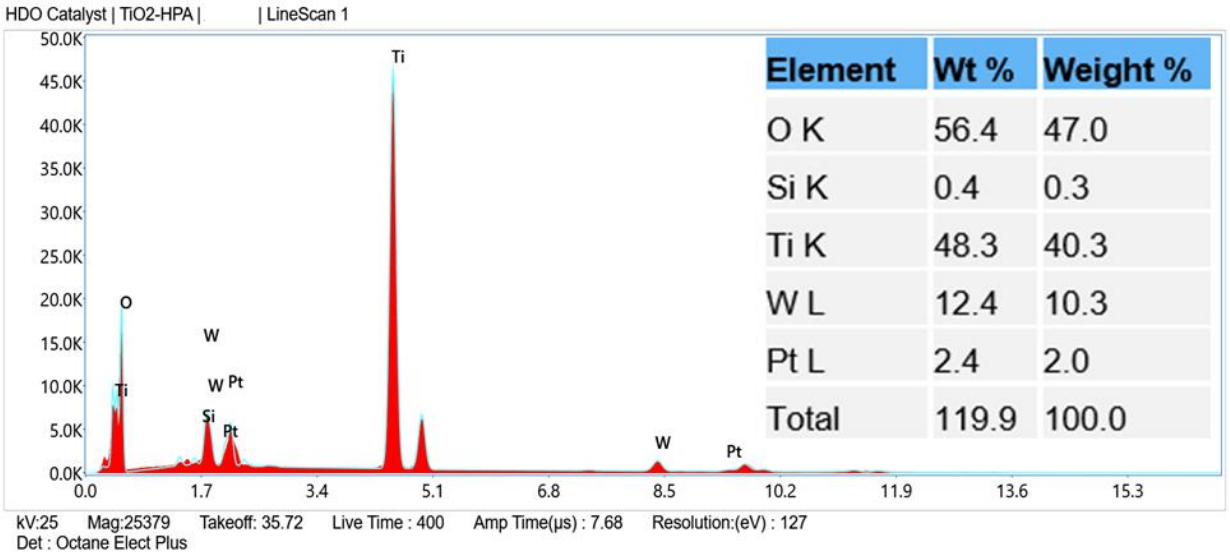

Element	Wt %	Weight %
O K	56.4	47.0
Si K	0.4	0.3
Ti K	48.3	40.3
W L	12.4	10.3
Pt L	2.4	2.0
Total	119.9	100.0

Fig. S2: Elemental composition of the line scan in Fig S1.

EDS line scan revealed a concentration of 2.4 wt. % Pt on the surface of the fibers as indicated by the figures S1 and S2 above. EDS line scans provide detailed information on elemental concentration across the fibers, here we notice a consistent distribution indicated by the line scan in figure S1. Measurements under accelerating voltage of 20 kV.

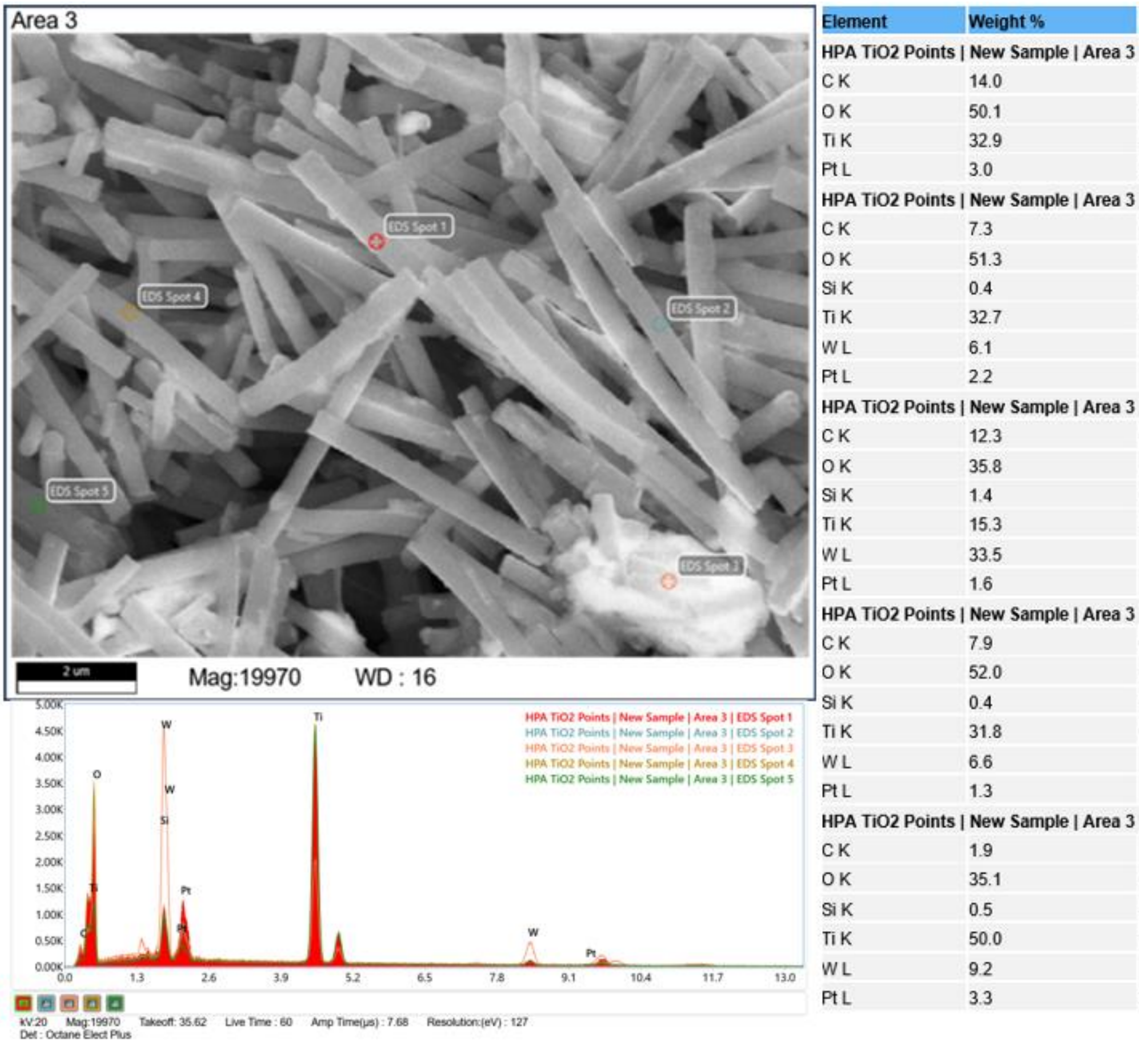

<table>
<tr><th>Element</th><th>Weight %</th></tr>
<tr><td colspan="2">HPA TiO2 Points | New Sample | Area 3</td></tr>
<tr><td>C K</td><td>14.0</td></tr>
<tr><td>O K</td><td>50.1</td></tr>
<tr><td>Ti K</td><td>32.9</td></tr>
<tr><td>Pt L</td><td>3.0</td></tr>
<tr><td colspan="2">HPA TiO2 Points | New Sample | Area 3</td></tr>
<tr><td>C K</td><td>7.3</td></tr>
<tr><td>O K</td><td>51.3</td></tr>
<tr><td>Si K</td><td>0.4</td></tr>
<tr><td>Ti K</td><td>32.7</td></tr>
<tr><td>W L</td><td>6.1</td></tr>
<tr><td>Pt L</td><td>2.2</td></tr>
<tr><td colspan="2">HPA TiO2 Points | New Sample | Area 3</td></tr>
<tr><td>C K</td><td>12.3</td></tr>
<tr><td>O K</td><td>35.8</td></tr>
<tr><td>Si K</td><td>1.4</td></tr>
<tr><td>Ti K</td><td>15.3</td></tr>
<tr><td>W L</td><td>33.5</td></tr>
<tr><td>Pt L</td><td>1.6</td></tr>
<tr><td colspan="2">HPA TiO2 Points | New Sample | Area 3</td></tr>
<tr><td>C K</td><td>7.9</td></tr>
<tr><td>O K</td><td>52.0</td></tr>
<tr><td>Si K</td><td>0.4</td></tr>
<tr><td>Ti K</td><td>31.8</td></tr>
<tr><td>W L</td><td>6.6</td></tr>
<tr><td>Pt L</td><td>1.3</td></tr>
<tr><td colspan="2">HPA TiO2 Points | New Sample | Area 3</td></tr>
<tr><td>C K</td><td>1.9</td></tr>
<tr><td>O K</td><td>35.1</td></tr>
<tr><td>Si K</td><td>0.5</td></tr>
<tr><td>Ti K</td><td>50.0</td></tr>
<tr><td>W L</td><td>9.2</td></tr>
<tr><td>Pt L</td><td>3.3</td></tr>
</table>

Fig. S3: Concentration of Pt across different spots of the catalytic scaffold. Low concentrations of 1.3 wt. % and high 3.3 wt. % is observed.

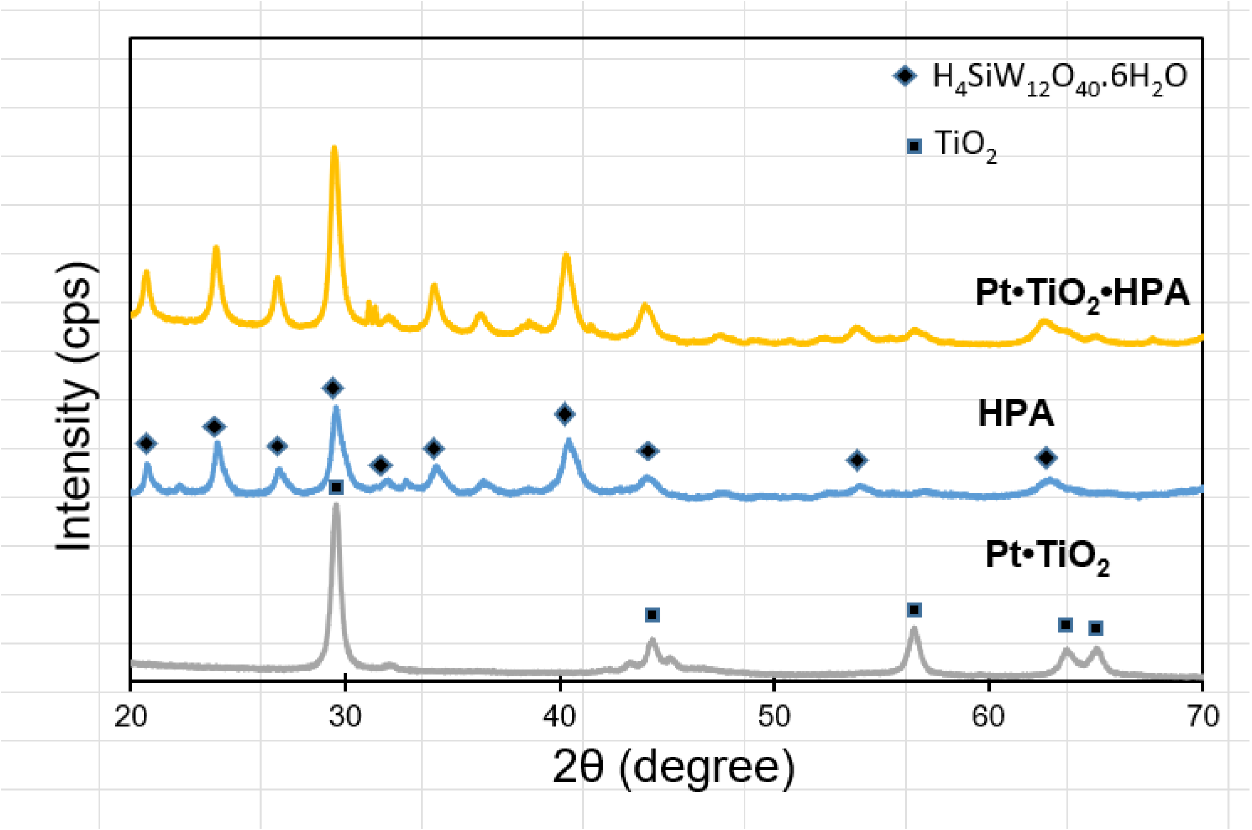

Fig. S4: XRD diffractogram for purchased tungstosilicic acid (HPA), electrospun Pt-TiO_2 fibers, and the final Pt-TiO_2-HPA catalyst. The Pt-TiO_2 fibers matched those of crystalline anatase.

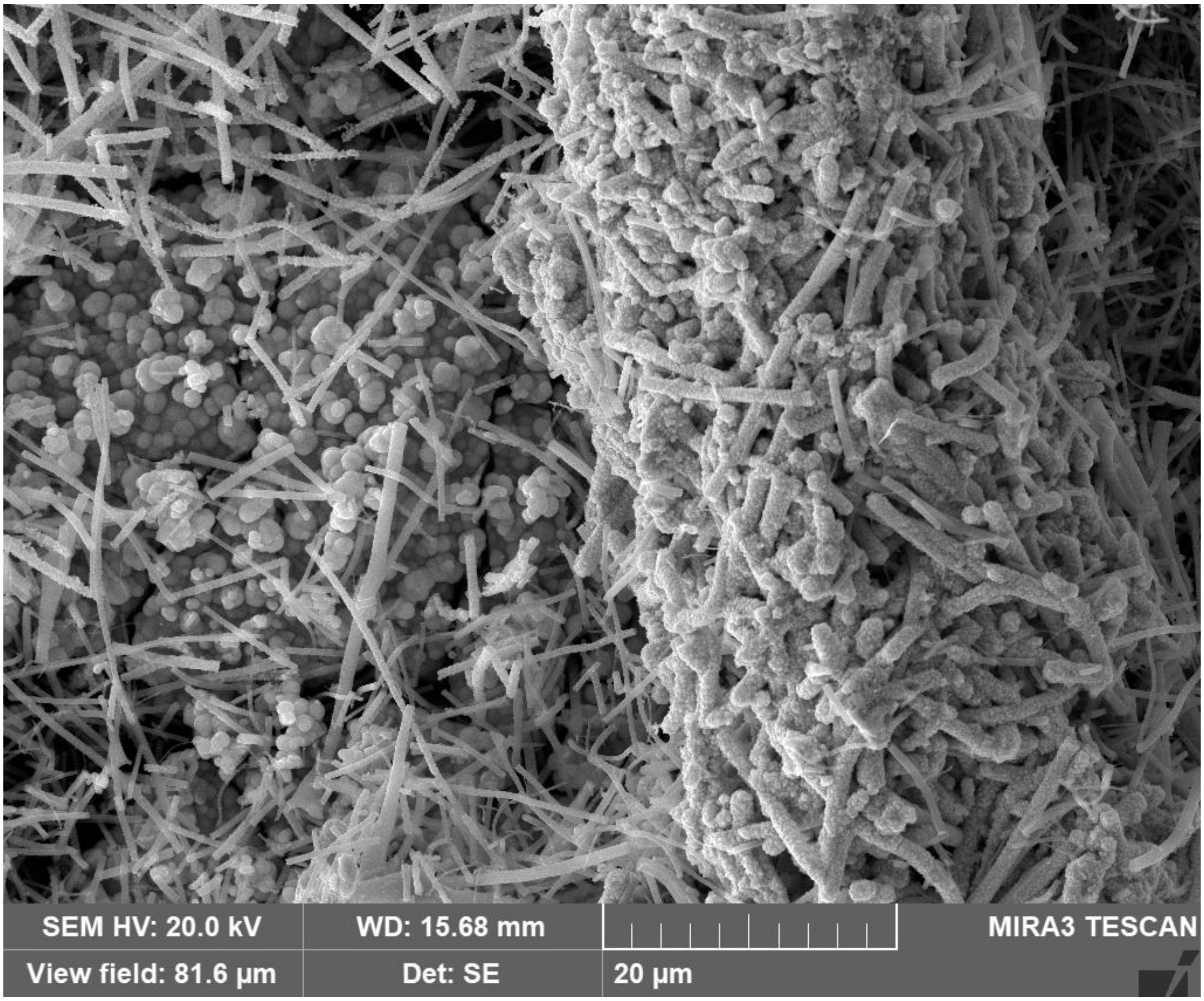

Fig. S5: Accumulation of reaction debris on the catalytic fiber after HDO.

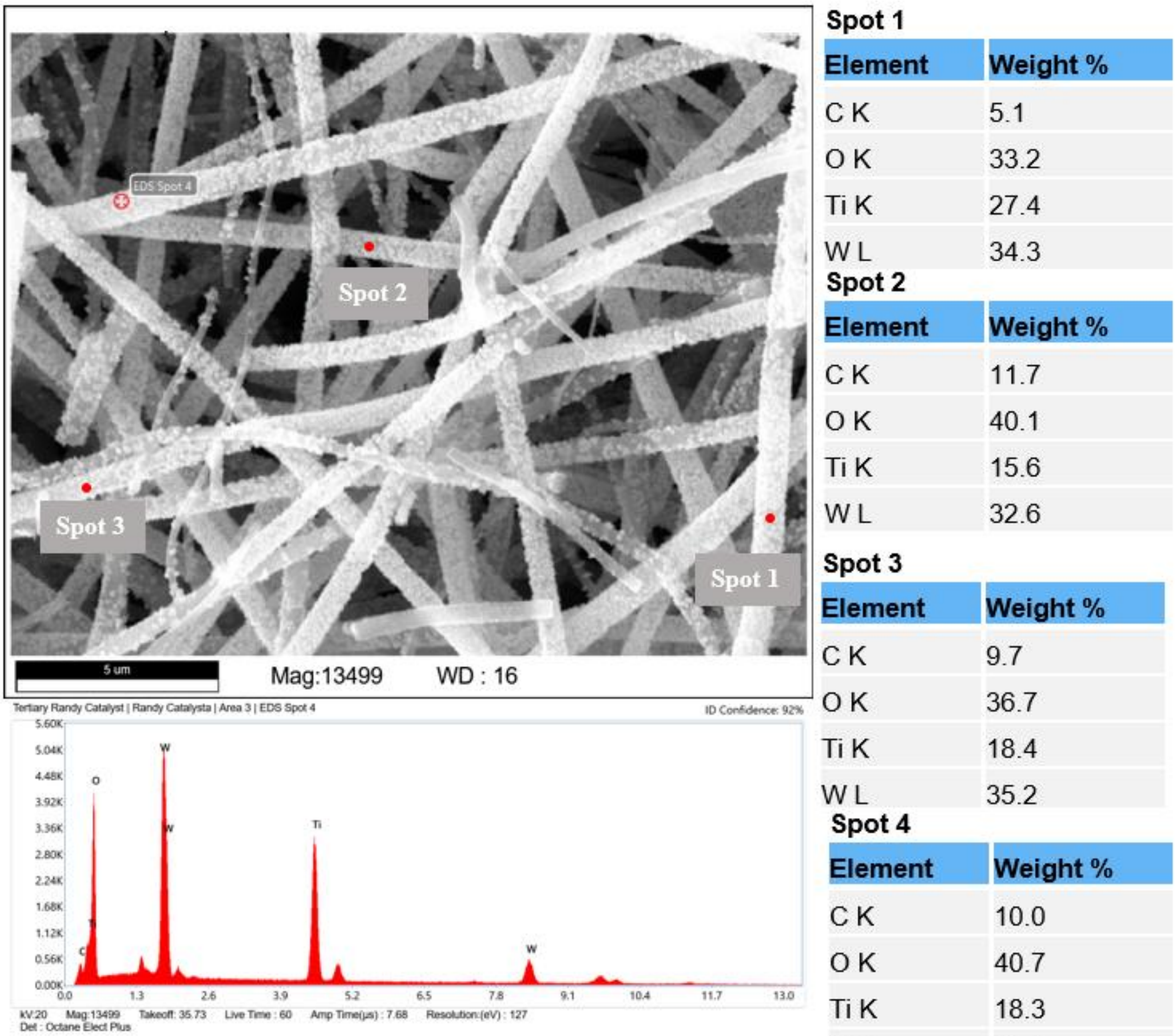

Spot 1

Element	Weight %
C K	5.1
O K	33.2
Ti K	27.4
W L	34.3

Spot 2

Element	Weight %
C K	11.7
O K	40.1
Ti K	15.6
W L	32.6

Spot 3

Element	Weight %
C K	9.7
O K	36.7
Ti K	18.4
W L	35.2

Spot 4

Element	Weight %
C K	10.0
O K	40.7
Ti K	18.3
W L	31.0

Fig. S6: Elemental composition of the spend catalyst. Pt was not detected on scanned spots. Carbon content was high ~10 wt. % suggesting possible presence of organics from the reaction process.

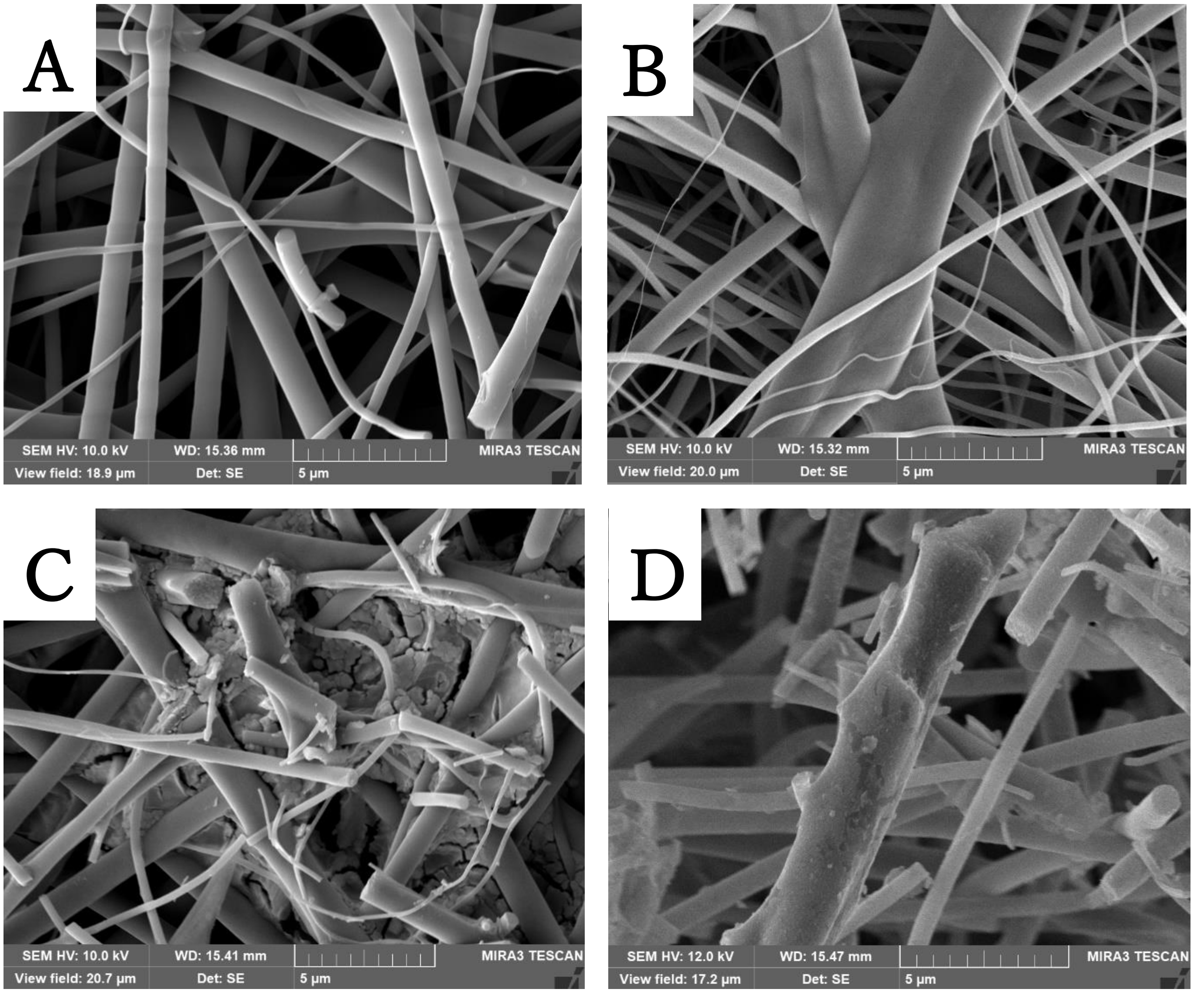

Fig. S7: Fabrication of catalytic fibers with final electroless deposition using a sol gel containing Pt precursors. (A) TiO_2-PVP fibers (B) TiO_2 fibers, (C) TiO_2-HPA fibers and (D) TiO_2-HPA-Pt fibers.

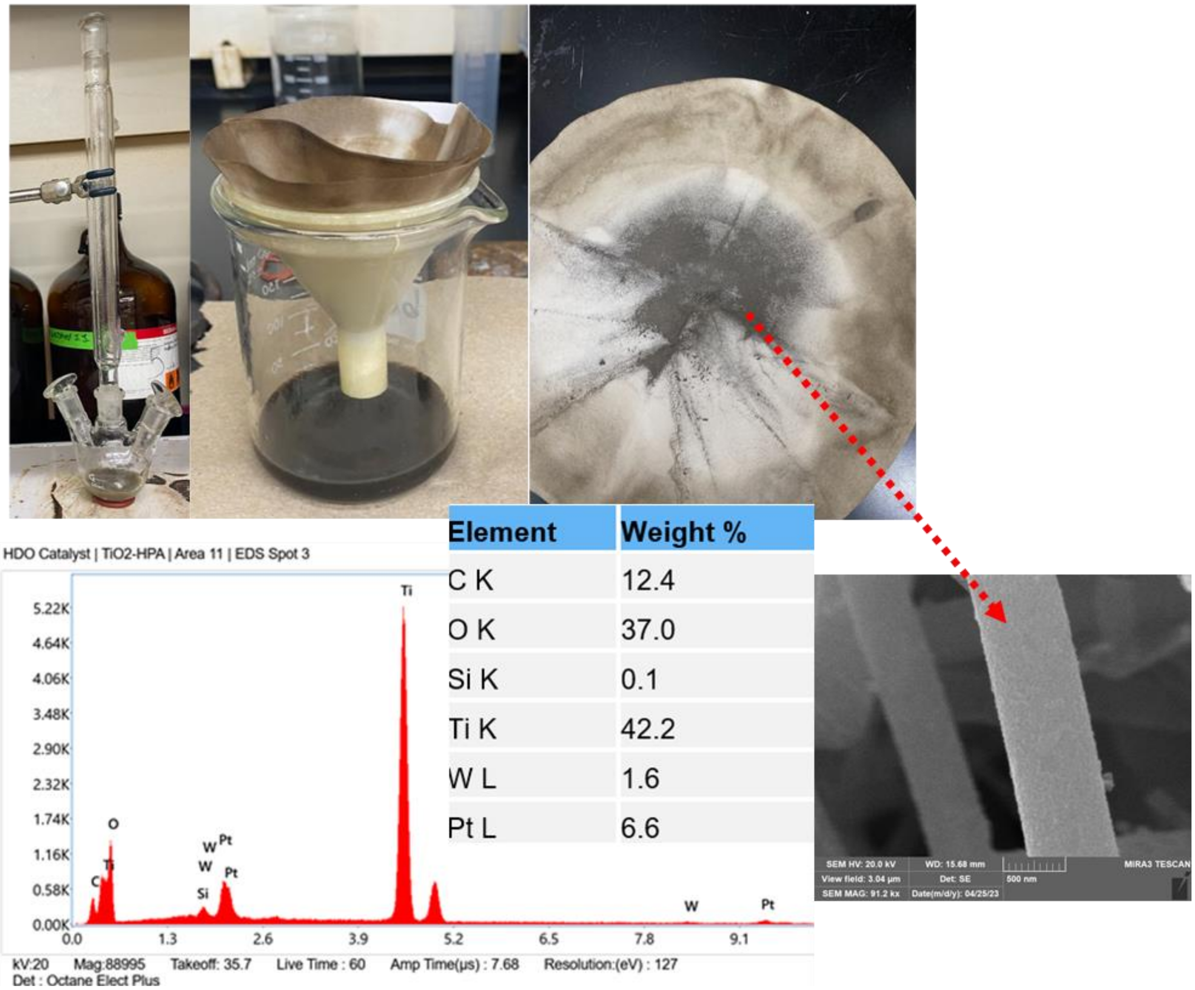

Element	Weight %
C K	12.4
O K	37.0
Si K	0.1
Ti K	42.2
W L	1.6
Pt L	6.6

Fig. S8: Electroless deposition of Pt NPs on TiO2 fibers coated with HPA. The final fiber surfaces had higher Pt concentrations ranging from 6.6 – 8.1 wt. %.

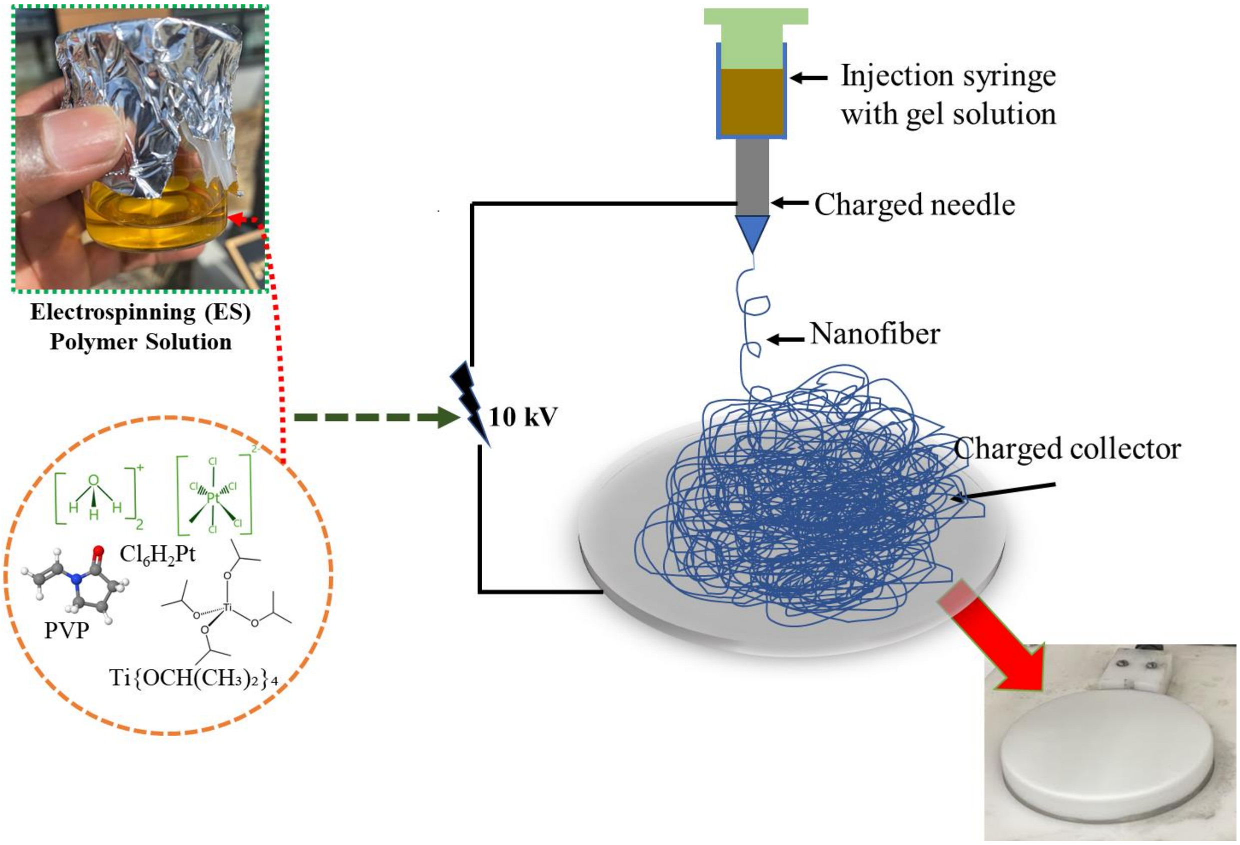

Fig. S9: Electrospinning process with electrospinning gel precursors.

The Pt-TiO2 catalyst was prepared in one step ES process where solution precursors were mixed at room temperature over 12 h. The final product was oxidized in air overnight followed by calcination in inert nitrogen atmosphere at 550 °C for 3 hours at a 2 °C/min ramp rate. The subsequent processes (**Fig. S10**) included HPA doping followed by drying and calcination at 300 °C to bond the catalyst components.

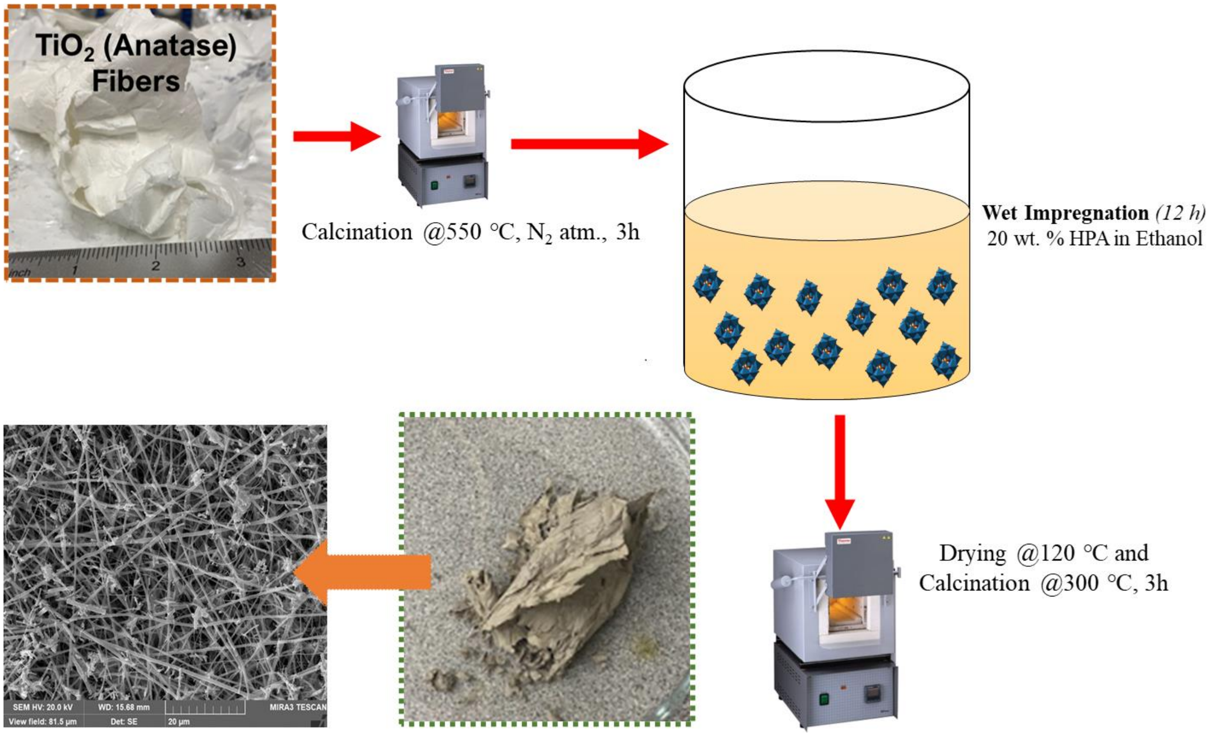

Fig. S10: Processing of the Pt-TiO_2-HPA catalyst.
